# Revisiting Entanglement within the Bohmian Approach to Quantum Mechanics

**DOI:** 10.3390/e20060473

**Published:** 2018-06-18

**Authors:** Claudia Zander, Angel Ricardo Plastino

**Affiliations:** 1 Physics Department, University of Pretoria, Pretoria 0002, South Africa; 2 CeBio y Secretaria de Investigaciones, Universidad Nacional del Noroeste de la Prov. de Buenos Aires-UNNOBA y CONICET, Roque Saenz Peña 456,6000 Junín, Argentina

**Keywords:** Bohmian dynamics, entanglement indicators, linear entropy

## Abstract

We revisit the concept of entanglement within the Bohmian approach to quantum mechanics. Inspired by Bohmian dynamics, we introduce two partial measures for the amount of entanglement corresponding to a pure state of a pair of quantum particles. One of these measures is associated with the statistical correlations exhibited by the joint probability density of the two Bohmian particles in configuration space. The other partial measure corresponds to the correlations associated with the phase of the joint wave function, and describes the non-separability of the Bohmian velocity field. The sum of these two components is equal to the total entanglement of the joint quantum state, as measured by the linear entropy of the single-particle reduced density matrix.

## 1. Introduction

The de Broglie-Bohm approach to quantum mechanics [1,2,3,4], also referred to as the pilot-wave theory, or the quantum theory of motion, has been, since the publication of the seminal works by Bohm, a subject of constant interest in the field of the foundations of quantum mechanics. Indeed, there has been a sustained research activity on the de Broglie-Bohm formulation along the years [5,6,7,8,9,10,11,12,13,14,15,16,17,18,19,20,21,22,23,24,25,26,27,28,29]. In an article reviewing and celebrating the first 100 years of quantum physics, Tegmark and Wheeler included the formulation of Bohmian mechanics within the list of the most significant events in the development of this field of Science [30]. The Bohmian approach has provided stimulating new perspectives on several fundamental aspects of quantum physics, among which we can mention the quantum measurement problem [5,6,7], quantum chaos [8], entanglement [9,31,32], the thermal equilibrium of quantum systems [10], the concept of quantum work [11], quantum cosmology [12,13,14,15], quantum gravity [16], and quantum chemistry (in the latter case, both at the practical [17,18] and at the conceptual-philosophical [19] levels). The Bohmian point of view also constitutes the starting point of possible extensions of quantum theory, as the intriguing proposal made by Valentini illustrates, leading to specific quantitative astrophysical and cosmological predictions that might be within the reach of observational tests [20,21]. The formalism of Bohm theory has also been applied to the treatment of problems in thermal physics, such as the classical Hamilton-Jacobi formulation of Fourier heat conduction [22].

In Bohm’s model of quantum mechanics the particles constituting a physical system have well defined positions in configuration space. The full description of the system is given by the particles’ configuration (i.e., the particles’ positions) and by a many-particle wave function. The particles’ configuration evolution is determined by the wave function through a “guiding” equation, while the wave function evolves according to the standard many-particle Schrödinger equation. Even though the particles are assumed to have, at each time, well defined positions, knowledge about these positions is not accessible. All we can know about these positions is their probability density distribution, given by the squared modulus of the wave function. The position observable plays a dominant role within the Bohmian formulation. In particular, within this approach, the outcome of an experiment in which any physical observable is measured is registered by the final configuration of the particles of the experimental apparatus. The Bohmian model thus highlights a basic feature of any measurement process, which is that “... all experiments and certainly all measurements in physics are in the last analysis essentially kinematic, for they are ultimately based on observations of the position of a particle or of a pointer on a scale as a function of time” [33]. Other interesting recent approaches to the foundations of quantum mechanics, such as the entropic dynamics formulation proposed by Caticha [34], also stress the special role played by the position observable. It is worth emphasizing that Bohm’s formulation of the quantum measurement process is fully consistent with Born’s rule in standard quantum mechanics and, consequently, the experimental predictions of Bohm’s theory coincide with those of the usual quantum mechanical formalism (see, however, Valentini’s Bohm-based proposal for an extension of quantum theory [20,21]). The basic quantal non-locality is explicitly expressed in the de Broglie-Bohm formalism. In point of fact, the Bell inequalities were inspired by Bell’s reaction to the work of Bohm [35].

In spite of the intense research work that has been devoted to Bohmian dynamics and its applications, relatively little attention has been paid to the quantitative analysis of entanglement within the Bohmian approach. The aim of the present contribution is to advance two quantitative indicators of the entanglement between two Bohmian particles. These quantities are explicitly formulated in terms of the Bohmian formalism. One of them corresponds to the statistical correlations exhibited by the joint probability density of the positions of the two particles. The other one is a quantitative measure of the non-separability of the Bohmian flow in the two-particle configuration space. We explore the main properties of these indicators and, as an illustrative application, we use these measures to investigate the decoherence-like process associated with two particles evolving under the effect of quantum friction.

The paper is organized as follows. In Section 2 we provide a brief review of the Bohmian theory. In Section 3 we introduce two quantitative indicators of entanglement within the Bohmian approach to quantum physics. In Section 4 we apply these measures to a system of two particles evolving under quantum friction. Finally, some conclusions are drawn in Section 5.

## 2. Bohmian Formulation of Quantum Dynamics

Bohmian dynamics includes, as one of its components, most of the formal apparatus of standard quantum mechanics. Indeed, a Bohmian quantum particle is endowed with a wave function ψ(r,t) governed by the Schrödinger equation. On top of this, the Bohmian particle has a definite position r that evolves in time according to the classical equation $\frac{dr}{dt}=\frac{p}{m}=v$, where p and v respectively denote the particle’s linear momentum and velocity. The position r of a Bohmian particle is the paradigmatic example of a hidden variable in a quantum theory. Within the Bohmian formulation it is assumed that the result of a position measurement is predetermined, even though it is not predictable. This state of affairs propagates to any other kind of physical measurement, since all of them translate, at some stage, into the position of some particles in the measuring device [34,35].

In spite of its classical flavor, there are fundamental differences between Bohmian dynamics and standard classical dynamics. In contrast to what happens in classical mechanics, the velocity **v** and the linear momentum p are not free variables anymore. They are instead determined, through the wave function ψ(r,t), by the particle’s position r(t) at each time *t*. The particle moves according to a first order differential equation,

(1) $$\frac{dr}{dt}=v\left(r,t\right),$$

with the flow in configuration space given by the velocity field v(r,t), determined by

(2) $$v\left(r,t\right)=-\frac{i\hbar }{2m}\left[\frac{1}{\psi (r,t)}\nabla \left(\psi \left(r,t\right)\right)-\frac{1}{\psi^{*}\left(r,t\right)}\nabla \left(\psi^{*}\left(r,t\right)\right)\right],$$

where ψ(r,t) is a time-dependent solution of Schrödiner equation. When preparing a state of the particle, one neither has control over, nor knowledge of, the particular initial value adopted by r. In this regard, the only accessible knowledge consists of the probability density ρ(r,t) corresponding to the different possible particle’s positions, given by

(3) $$\rho \left(r,t\right)={\left|\psi \left(r,t\right)\right|}^{2}.$$

Associated with the configuration space flow (1) there is a Liouville-like continuity equation for the probability density ρ(r,t),

(4) $$\frac{\partial \rho }{\partial t}+\nabla \cdot \left(v\rho \right)=0.$$

The form (2) of the velocity field, together with the Schrödinger equation and the continuity Equation (4), have an important consequence: if the probability density of initial positions of the Bohmian particle satisfies the relation (3), then it also satisfies this relation at all later times.

## 3. Entanglement Between Two Bohmian Particles: Configuration and Phase Entanglement

In this section, we are going to introduce two quantitative entanglement indicators, closely related to the main features of the Bohmian approach, for pure states of a pair of quantum particles. We are going to consider two spinless quantum particles moving in one spatial dimension (the extension of the present developments to *D* dimensions is straightforward) with coordinates denoted by $x_{1}$ and $x_{2}$. In terms of the standard quantum mechanical formalism, the two quantum particles are described by the pure state

(5) $$|\psi \rangle =\int dx_{1}dx_{2}\psi \left(x_{1},x_{2}\right)|x_{1}\rangle |x_{2}\rangle ,$$

where $|x_{1}x_{2}\rangle =|x_{1}\rangle |x_{2}\rangle$, $\psi \left(x_{1},x_{2}\right)=\langle x_{1},x_{2}|\psi \rangle$ and $|x_{i}\rangle$ is an eigenstate of the *i*-particle position operator.

An important aspect of Bohm’s approach is the assumption that at each point in time particles have well defined positions and, consequently, describe well defined orbits in configuration space, although the initial conditions associated with these orbits are not experimentally controllable. At the level of individual orbits of the pair of Bohmian particles that we are discussing here, quantum entanglement manifest itself by the fact that the velocity field $v(x_{1},x_{2})$ is not separable. That is, $v\left(x_{1},x_{2}\right)\neq \left(v_{1}\left(x_{1}\right),v_{2}\left(x_{2}\right)\right)$. In other words, each of the two components $\left(v_{1},v_{2}\right)$ of the vector field v depends, in general, on both particles’ coordinates $\left(x_{1},x_{2}\right)$. This means that the behaviors of both particles are intertwined. Roughly speaking, one can say that each particle affects the behavior of the other one, even if there is no interaction potential involved, and the particles are far apart. This state of affairs is highly counter-intuitive and has been the focus of considerable attention in the literature. In fact, virtually all discussions of entanglement within the Bohmian approach have dealt, in one way or another, with this aspect of Bohmian dynamics (see, for instance [31,32] and references therein). However, and in spite of its great theoretical-philosophical interest, the study of individual Bohmian trajectories does not lend itself to a quantitative characterization of the amount of entanglement associated with a two-particle system at a given time. In point of fact, at a given instant *t*, two Bohmian particles following an individual trajectory are located at a specific point in configuration space with coordinates $\left(x_{1}\left(t\right),x_{2}\left(t\right)\right)$, where the velocity field is given by a specific vector $v(x_{1},x_{2})$. Now, it is not possible to assess the amount of non-separability that a vector field has at a particular location $\left(x_{1},x_{2}\right)$. The non-separability of a vector field is a global property that is associated with a region of configuration space. A sensible quantitative indicator of this non-separability can then be given by an average value, evaluated over such a region. In addition, Bohm’s theory does involve a probability density in configuration space: the probability density $\rho \left(x_{1},x_{2}\right)={\left|\psi \left(x_{1},x_{2}\right)\right|}^{2}$ of having the Bohmian particles at different locations. Consequently, it is reasonable to expect that an appropriate quantitative measure of the degree of non-separability of the Bohmian velocity field should be some sort of spatial average of non-sperability, related to the configuration space density $\rho (x_{1},x_{2})$ associated with the particles’ positions. We thus see that it seems inevitable that the probability density in configuration space has to be involved in a quantitative treatment of entanglement within Bohmian mechanics. Now, once this density has been incorporated to the discussion, there appears another contribution to entanglement that has to be taken into account, which is the (classical-like) statistical correlations present in the configuration space probability density itself. Mathematically, these correlations are given by the non-factorizability of the density, $\rho \left(x_{1},x_{2}\right)\neq \rho_{1}\left(x_{1}\right)\rho_{2}\left(x_{2}\right)$. On the basis of these considerations we are going to propose two indicators of the amount of entanglement of two particles at a given time, that provide quantitative measures of the above mentioned aspects of Bohmian mechanics: the non-separability of the velocity field and the classical-like correlations of the probability density in configuration space. It is worth stressing that, even though we are not going to refer explicitly to individual Bohmian orbits, our entanglement indicators are directly related to the above explained essential aspects of the Bohmian approach which originate, in turn, from the assumption that individual orbits exists. We can make here an analogy with the Gibbs approach to classical statistical mechanics. When using the canonical statistical ensemble to describe a system at thermal equilibrium one does not refer explicitly to individual orbits of a Hamiltonian system. However, it is clear that Hamiltonian dynamics still plays a fundamental role in, and is indeed at the foundations of, the canonical formulation of statistical mechanics.

The density matrix corresponding to the pure state (5) is

(6) $$\hat{\rho }=\left|\psi \rangle \langle \psi \right|=\int dx_{1}dx_{2}dx_{1}^{\prime }dx_{2}^{\prime }\psi \left(x_{1},x_{2}\right)\psi^{*}\left(x_{1}^{\prime },x_{2}^{\prime }\right)|x_{1}x_{2}\rangle \langle x_{1}^{\prime }x_{2}^{\prime }|$$

and has matrix elements

(7) $$\langle x_{1}x_{2}|\hat{\rho }|x_{1}^{\prime }x_{2}^{\prime }\rangle =\psi \left(x_{1},x_{2}\right)\psi^{*}\left(x_{1}^{\prime },x_{2}^{\prime }\right).$$

The density matrix $\hat{\rho }$ should not be confused with the spatial density $\rho (x_{1},x_{2})$ mentioned before. The spatial density corresponds to the diagonal elements of the operator $\hat{\rho }$, that is $\rho \left(x_{1},x_{2}\right)=\langle x_{1}x_{2}|\hat{\rho }|x_{1}x_{2}\rangle$. The marginal density matrix ${\hat{\rho }}_{1}={\mathrm{Tr}}_{2}\hat{\rho }$ describing particle 1, has matrix elements

(8) $$\begin{array}{ccc} \langle x_{1}|{\hat{\rho }}_{1}|x_{2}\rangle & = & \int \langle x_{1}x_{3}|\hat{\rho }|x_{2}x_{3}\rangle dx_{3}\\ & = & \int \psi \left(x_{1},x_{3}\right)\psi^{*}\left(x_{2},x_{3}\right)dx_{3}. \end{array}$$

The linear entropy of ${\hat{\rho }}_{1}$ constitutes a useful measure or indicator of the amount of entanglement of the state ψ,

(9) $$E=1-\mathrm{Tr}({\hat{\rho }}_{1}^{2}),$$

where one has,

(10) $$\begin{array}{ccc} \mathrm{Tr}({\hat{\rho }}_{1}^{2}) & = & \int \langle x_{1}|{\hat{\rho }}_{1}|x_{2}\rangle \langle x_{2}|{\hat{\rho }}_{1}|x_{1}\rangle dx_{1}dx_{2}\\ & = & \int \psi \left(x_{1},x_{3}\right)\psi^{*}\left(x_{2},x_{3}\right)\psi^{*}\left(x_{1},x_{4}\right)\psi \left(x_{2},x_{4}\right)dx_{1}dx_{2}dx_{3}dx_{4}. \end{array}$$

Alternatively, we can express this entanglement indicator in terms of the marginal density matrix ${\hat{\rho }}_{2}={\mathrm{Tr}}_{1}\hat{\rho }$ corresponding to particle 2, so one has

(11) $$E=1-\mathrm{Tr}\left({\hat{\rho }}_{1}^{2}\right)=1-\mathrm{Tr}\left({\hat{\rho }}_{2}^{2}\right).$$

The quantity E constitutes a useful, practical way to assess quantitatively the amount of entanglement exhibited by a two-particle pure state. In fact, it has been applied to the study of quantum entanglement in many different settings (see, for instance, [36]) due to its various computational advantages, both from the analytical and from the numerical points of view. However, this quantity does not have a clear interpretation in terms of the Bohmian theory. Our aim, inspired by the Bohmian approach to quantum mechanics, is to decompose the entanglement indicator E into two parts having a clear meaning in terms of the two basic ingredients of the dynamics of two Bohmian particles: their joint probability density in configuration space, and the joint velocity field describing the probability density flow. The above mentioned two contributions to the total entanglement, as measured by E, constitute quantitative indicators of entanglement that we shall respectively call configuration entanglement $E_{c}$, and phase entanglement, $E_{p}$.

Following the Bohmian approach, we express the wave function as

(12) $$\psi \left(x_{1},x_{2}\right)=R^{\frac{1}{2}}\left(x_{1},x_{2}\right)e^{i\alpha (x_{1},x_{2})},$$

where *R* and α are both real functions, and R≥0. The quantity $R\left(x_{1},x_{2}\right)={\left|\psi \left(x_{1},x_{2}\right)\right|}^{2}$ satisfies a continuity equation and represents the joint probability density of the two Bohmian particles in configuration space. The wave function (12) can be entangled through $R(x_{1},x_{2})$, through the phase $\alpha (x_{1},x_{2})$, or through both these quantities. Entanglement through the density *R* means that the probability density $R(x_{1},x_{2})$ cannot be factorized as $R\left(x_{1},x_{2}\right)=R_{1}\left(x_{1}\right)R_{2}\left(x_{2}\right)$. This lack of factorizability, which we refer to as “configuration entanglement” corresponds to a correlation (in the classical sense) of the probability density $R(x_{1},x_{2})$. On the other hand, entanglement through the phase $\alpha (x_{1},x_{2})$ means that it cannot be additively decomposed as $\alpha \left(x_{1},x_{2}\right)=\alpha_{1}\left(x_{1}\right)+\alpha_{2}\left(x_{2}\right)$. This lack of additive decomposability means that the Bohmian dynamical equations of particles 1 and 2 are not independent. To clarify this last point, lets consider the equations of motion of the two Bohmian particles,

(13) $$\begin{array}{ccc} \frac{dx_{1}}{dt} & = & v_{1}\left(x_{1},x_{2}\right)=\frac{\hbar }{m}\frac{\partial \alpha }{\partial x_{1}},\\ \frac{dx_{2}}{dt} & = & v_{2}\left(x_{1},x_{2}\right)=\frac{\hbar }{m}\frac{\partial \alpha }{\partial x_{2}}. \end{array}$$

In general, if $\alpha \left(x_{1},x_{2}\right)\neq \alpha_{1}\left(x_{1}\right)+\alpha_{2}\left(x_{2}\right)$, the two ordinary differential equations in (13) are coupled to each other. On the contrary, if $\alpha \left(x_{1},x_{2}\right)\neq \alpha_{1}\left(x_{1}\right)+\alpha_{2}\left(x_{2}\right)$, these two equations of motion decouple, and one has,

(14) $$\begin{array}{ccc} \frac{dx_{1}}{dt} & = & v_{1}\left(x_{1}\right)=\frac{\hbar }{m}\frac{d\alpha_{1}}{dx_{1}},\\ \frac{dx_{2}}{dt} & = & v_{2}\left(x_{2}\right)=\frac{\hbar }{m}\frac{d\alpha_{2}}{dx_{2}}. \end{array}$$

That is, in the latter case the equations of motion of the two Bohmian particles separate into two independent equations.

In the following two Subsections we are going to propose two quantitative indicators for configuration entanglement, and for phase entanglement, and determine some of their basic properties.

### 3.1. Configuration Entanglement

As a quantitative indicator of entanglement we propose,

(15) $$E_{c}=1-\int dx_{1}dx_{2}dx_{3}dx_{4}\sqrt{R\left(x_{1},x_{3}\right)R\left(x_{2},x_{3}\right)R\left(x_{1},x_{4}\right)R\left(x_{2},x_{4}\right)}.$$

The quantity $E_{c}$ can be interpreted as an indicator of classical correlations in the probability density $R(x_{1},x_{2})$. Indeed, if this density is factorizable, $R\left(x_{1},x_{2}\right)=R_{1}\left(x_{1}\right)R_{2}\left(x_{2}\right)$ we have,

(16) $$\begin{array}{c} \int dx_{1}dx_{2}dx_{3}dx_{4}\sqrt{R\left(x_{1},x_{3}\right)R\left(x_{2},x_{3}\right)R\left(x_{1},x_{4}\right)R\left(x_{2},x_{4}\right)}\\ =\int dx_{1}dx_{2}dx_{3}dx_{4}R_{1}\left(x_{1}\right)R_{1}\left(x_{2}\right)R_{2}\left(x_{3}\right)R_{2}\left(x_{4}\right)=1 \end{array}$$

due to normalization. Therefore, in the case that $R(x_{1},x_{2})$ is factorizable we have $E_{c}=0$. Now, we have

(17) $$\begin{array}{c} \int dx_{1}dx_{2}dx_{3}dx_{4}\sqrt{R\left(x_{1},x_{3}\right)R\left(x_{2},x_{3}\right)R\left(x_{1},x_{4}\right)R\left(x_{2},x_{4}\right)}\\ =\int dx_{1}dx_{2}\left\{\int dx_{3}\sqrt{R\left(x_{1},x_{3}\right)R\left(x_{2},x_{3}\right)}\right\}\left\{\int dx_{4}\sqrt{R\left(x_{1},x_{4}\right)R\left(x_{2},x_{4}\right)}\right\}\\ =\int dx_{1}dx_{2}{\left\{\int dx_{3}\sqrt{R\left(x_{1},x_{3}\right)R\left(x_{2},x_{3}\right)}\right\}}^{2}\\ \leq \int dx_{1}dx_{2}\left\{\int dx_{3}R\left(x_{1},x_{3}\right))\right\}\left\{\int dx_{4}R\left(x_{2},x_{4}\right)\right\}\\ =\int dx_{1}dx_{2}dx_{3}dx_{4}R\left(x_{1},x_{3}\right)R\left(x_{2},x_{4}\right)\\ =1. \end{array}$$

The inequality in (17) is due to the Schwartz inequality and the final equality is due to the normalization of $R(x_{1},x_{2})$. It follows from (15) and (17) that the configuration entanglement $E_{c}$ is always bounded according to

(18) $$0\leq E_{c}\leq 1,$$

achieving its lowest bound (that is, vanishing) when the joint probability density in configuration space is factorizable.

### 3.2. Phase Entanglement

As a quantitative indicator of the amount of phase entanglement we propose,

(19) $$\begin{array}{c} E_{p}=\int dx_{1}dx_{2}dx_{3}dx_{4}\left\{1-\exp\left[i\left\{\alpha \left(x_{1},x_{3}\right)-\alpha \left(x_{2},x_{3}\right)-\alpha \left(x_{1},x_{4}\right)+\alpha \left(x_{2},x_{4}\right)\right\}\right]\right\}\times \\ \times R^{\frac{1}{2}}\left(x_{1},x_{3}\right)R^{\frac{1}{2}}\left(x_{2},x_{3}\right)R^{\frac{1}{2}}\left(x_{1},x_{4}\right)R^{\frac{1}{2}}\left(x_{2},x_{4}\right). \end{array}$$

We have that

(20) $$\begin{array}{ccc} E_{p} & = & \left\{\int dx_{1}dx_{2}dx_{3}dx_{4}\left|\psi \left(x_{1},x_{3}\right)\psi^{*}\left(x_{2},x_{3}\right)\psi^{*}\left(x_{1},x_{4}\right)\psi \left(x_{2},x_{4}\right)\right|\right\}\\ & & -\left|\left\{\int dx_{1}dx_{2}dx_{3}dx_{4}\psi \left(x_{1},x_{3}\right)\psi^{*}\left(x_{2},x_{3}\right)\psi^{*}\left(x_{1},x_{4}\right)\psi \left(x_{2},x_{4}\right)\right\}\right|\\ & \geq & 0. \end{array}$$

In this last equation we used the fact that

(21) $$\int dx_{1}dx_{2}dx_{3}dx_{4}\psi \left(x_{1},x_{3}\right)\psi^{*}\left(x_{2},x_{3}\right)\psi^{*}\left(x_{1},x_{4}\right)\psi \left(x_{2},x_{4}\right)=\mathrm{Tr}\left({\hat{\rho }}_{1}^{2}\right)$$

is always a real positive number. It also follows from (17) and (19) (remembering that $R^{\frac{1}{2}}\left(x_{1},x_{2}\right)=\left|\psi \left(x_{1},x_{2}\right)\right|$) that $E_{p}\leq 1$. Summing up, we have

(22) $$0\leq E_{p}\leq 1.$$

In the case that the phase $\alpha (x_{1},x_{2})$ is additively decomposable, $\alpha \left(x_{1},x_{2}\right)=\alpha_{1}\left(x_{1}\right)+\alpha_{2}\left(x_{2}\right)$, we have

(23) $$\begin{array}{c} \alpha \left(x_{1},x_{3}\right)-\alpha \left(x_{2},x_{3}\right)-\alpha \left(x_{1},x_{4}\right)+\alpha \left(x_{2},x_{4}\right)\\ =\alpha_{1}\left(x_{1}\right)+\alpha_{2}\left(x_{3}\right)-\alpha_{1}\left(x_{2}\right)-\alpha_{2}\left(x_{3}\right)-\alpha_{1}\left(x_{1}\right)-\alpha_{2}\left(x_{4}\right)+\alpha_{1}\left(x_{2}\right)+\alpha_{2}\left(x_{4}\right)=0, \end{array}$$

implying that $E_{p}=0$. On the other hand, if $E_{p}=0$ we must have that

(24) $$\exp\;\left[i\left\{\alpha \left(x_{1},x_{3}\right)-\alpha \left(x_{2},x_{3}\right)-\alpha \left(x_{1},x_{4}\right)+\alpha \left(x_{2},x_{4}\right)\right\}\right]=\exp\;\left[i\delta \right]$$

for some real constant δ. Therefore (assuming $\alpha (x_{1},x_{2})$ to be a continuous function) we have

(25) $$\alpha \left(x_{1},x_{3}\right)-\alpha \left(x_{2},x_{3}\right)-\alpha \left(x_{1},x_{4}\right)+\alpha \left(x_{2},x_{4}\right)=\delta .$$

This relation must hold for all values of $x_{1},x_{2},x_{3},x_{4}$. Therefore, fixing some constant values $x_{20}$ and $x_{40}$ for $x_{2}$ and $x_{4}$, respectively, we can write

(26) $$\alpha \left(x_{1},x_{3}\right)=\alpha \left(x_{1},x_{40}\right)+\alpha \left(x_{20},x_{3}\right)-\alpha \left(x_{20},x_{40}\right)+\delta .$$

Defining now

(27) $$\begin{array}{ccc} \alpha_{1}\left(x_{1}\right) & = & \alpha \left(x_{1},x_{40}\right)-\frac{1}{2}\alpha \left(x_{20},x_{40}\right)+\frac{\delta }{2}\\ \alpha_{2}\left(x_{3}\right) & = & \alpha \left(x_{20},x_{3}\right)-\frac{1}{2}\alpha \left(x_{20},x_{40}\right)+\frac{\delta }{2} \end{array}$$

we have

(28) $$\alpha \left(x_{1},x_{3}\right)=\alpha_{1}\left(x_{1}\right)+\alpha_{2}\left(x_{3}\right)$$

and therefore the function α is additively decomposable. In summary, $E_{p}=0$ if and only if α is additively decomposable.

Finally, it can be verified after some algebra that the total entanglement of the two-particle pure quantum state, as measured by E (given by (9)), is equal to the sum of the entanglement of the configuration and the phase contributions,

(29) $$E=E_{c}+E_{p}.$$

Please note that the bounds (18) and (22), respectively satisfied by the configuration and the phase entanglement, are consistent with the bounds 0≤E≤1 satisfied by the total entanglement E. For a factorizable quantum (pure) state of the two particles, both contributions $E_{c}$ and $E_{p}$ to the entanglement between the particles achieve their respective lower bounds (that is, both of them vanish). However, the configuration and the phase entanglements cannot both achieve their upper bounds (they cannot both be equal to 1) because the total entanglement satisfies $E_{c}+E_{p}\leq 1$. This means that a state with high configuration entanglement must have low phase entanglement, and vice versa.

## 4. Entanglement Dynamics and Quantum Friction

We shall now apply the configuration and phase quantitative entanglement indicators to explore the entanglement dynamics of two quantum particles evolving according to a nonlinear Schrödinger equation incorporating quantum friction effects. Nonlinear Schrödinger equations have attracted considerable attention in recent years, and have been applied to the description of diverse physical phenomena. Closely related to Bohmian dynamics is the nonlinear Schrödinger equation proposed by Nassar and Miret-Artés in [6],

(30) $$i\hbar \frac{\partial \psi (x,t)}{\partial t}=\left[H\left(x,t\right)+i\hbar \left(W_{c}\left(x,t\right)+W_{f}\left(x,t\right)\right)\right]\psi \left(x,t\right)$$

where $H=-\frac{\hbar^{2}}{2m}\frac{\partial^{2}}{\partial x^{2}}+V\left(x\right)$ is the standard Hamiltonian for a quantum particle of mass *m* moving in one dimension under the potential V(x). We also have,

(31) $$W_{c}\left(x,t\right)=-\kappa \left[{\ln\;|\psi \left(x,t\right)|}^{2}-{\langle \ln\;|\psi \left(x,t\right)|}^{2}\rangle \right],$$

and

(32) $$W_{f}\left(x,t\right)=-\frac{\nu }{2}\left[\ln\;\frac{\psi (x,t)}{\psi^{*}\left(x,t\right)}-\langle \ln\;\frac{\psi (x,t)}{\psi^{*}\left(x,t\right)}\rangle \right],$$

where

(33) $${\langle \ln\;|\psi \left(x,t\right)|}^{2}{\rangle =\int |\psi \left(x,t\right)|}^{2}\ln\;{\left|\psi \left(x,t\right)\right|}^{2}dx,$$

and

(34) $$\langle \ln\;\frac{\psi (x,t)}{\psi^{*}\left(x,t\right)}\rangle =\int {\left|\psi \left(x,t\right)\right|}^{2}\ln\;\left(\frac{\psi (x,t)}{\psi^{*}\left(x,t\right)}\right)\;\;dx.$$

In the above equations κ is a constant related to the resolution of position measurement and ν is a friction coefficient. The nonlinear wave Equation (30) was proposed as an effective description of the dynamics of a quantum particle undergoing a process of continuous position measurement including dissipation effects [6]. The non-linear logarithmic term $W_{c}$ advanced by Nassar in [5] was motivated by Mensky’s path integral formulation of continuous quantum measurements [37], whereas the term $W_{f}$ considered in [6] was inspired by Kostin’s work in connection with friction in quantum systems [38]. Several aspects of these kind of nonlinear evolution equations have been investigated in [39,40,41,42,43,44]. In terms of Bohmian dynamics, the friction term $W_{f}$ in the non-linear Schrödinger Equation (30) leads to a new term in the equations governing the evolution of the Bohmian velocity field, that can be interpreted as describing a drag force [6].

Please note that the friction effects described by the term $W_{f}$ in the nonlinear Schrödinger Equation (30) occur at the level of pure states. That is, the evolution of a quantum pure state is affected by these friction effects, but the state stays pure as it evolves. The presence of the term $W_{f}$ gives rise, for instance, to the decrease (dissipation) of the energy of the time-dependent state, but not to an increase of its entropy. Equation (30) is a Schrödinger-Langevin-like equation without the stochastic force term. These kind of equations have been the focus of considerable attention and applied to diverse problems [4,6]. Nonlinear wave equations like (30) can be regarded as incorporating phenomenological descriptions of friction that describe only some aspects of the dynamics of an open system (for instance, energy dissipation). In this sense, they differ from approaches based on master equations where, in general, the entropy of the system changes as it evolves. A classical analogue may contribute to clarify this situation. A classical conservative system, such as a standard harmonic oscillator, is both conservative and deterministic. Energy is conserved during evolution, and complete knowledge of the initial conditions (represented by a point in phase space) fully determines the state of the system at a later time (represented, again, by a point in phase space). In summary: a point in phase space deterministically evolves into another point in phase space. If we incorporate friction effects described by drag forces (for instance, of the form F=−av) the system is no longer conservative. Energy is not conserved. However, the system is still deterministic: a point in phase space still evolves into another well defined point in phase space. If one also incorporates stochastic forces (like in the Langevin equation) the system is no longer deterministic. Even if the initial conditions correspond to a point in phase space, to describe the evolution of the system one needs a time-dependent probability density in phase space. A quantum mechanical analogue of this situation is an open quantum system that evolves, for instance, according to the Linblad equation, and that has to be described by a time-dependent density matrix. On the other hand, a wave equation like (30) (or, more specifically, Equation (37) that we are going to consider later), governing the evolution of a quantum system that is at all times described by a pure state, can be regarded as a quantum mechanical analogue of a classical system that is affected by friction (drag forces) but is still deterministic. These classical systems have practical, conceptual, and historical relevance, and their properties have been the focus of investigation since long ago (a nice discussion can be found in Chapters 19 and 20 of [45]). Consequently, it is an interesting problem to explore their possible quantum mechanical counterparts.

We are now going to apply the configuration and phase entanglement indicators previously introduced, to explore the entanglement dynamics of two quantum particles governed by a particular two-dimensional version of the wave Equation (30), incorporating the friction term $W_{f}$, but not the nonlinear logarithmic term $W_{c}$. That is, in (30) we set κ=0. We consider the evolution of a two-dimensional Gaussian wave packet describing two (entangled) particles subjected to friction and moving in one spatial dimension in a common harmonic potential well. The Gaussian ansatz is

(35) $$\Psi \left(x_{1},x_{2},t\right)=e^{\lambda_{0}\left(t\right)+\lambda_{1}\left(t\right)x_{1}^{2}+\lambda_{2}\left(t\right)x_{2}^{2}+\lambda_{3}\left(t\right)x_{1}x_{2}+\lambda_{4}\left(t\right)x_{1}+\lambda_{5}\left(t\right)x_{2}},$$

where the coefficients $\lambda_{j}\left(t\right)$ are complex functions of time,

(36) $$\lambda_{j}\left(t\right)=\lambda_{jR}\left(t\right)+i\lambda_{jI}\left(t\right)\;\mathrm{for}\;j=0,\ldots ,5$$

with $\lambda_{jR},\lambda_{jI}\in R$. The $\lambda_{j}\left(t\right)$’s are then chosen so that (35) is a solution of a non-linear Schrödinger equation incorporating a friction term. This evolution equation is given by

(37) $$i\hbar \frac{\partial \Psi (x_{1},x_{2},t)}{\partial t}=\left[-\frac{\hbar^{2}}{2m}\left(\frac{\partial^{2}}{\partial x_{1}^{2}}+\frac{\partial^{2}}{\partial x_{2}^{2}}\right)+\frac{1}{2}m\omega^{2}\left(x_{1}^{2}+x_{2}^{2}\right)+i\hbar W_{f}\left(x_{1},x_{2},t\right)\right]\Psi \left(x_{1},x_{2},t\right),$$

where

(38) $$W_{f}\left(x_{1},x_{2},t\right)=-\frac{\nu }{2}\left[2i\alpha \left(x_{1},x_{2},t\right)-2i\int dx_{1}dx_{2}R\left(x_{1},x_{2},t\right)\alpha \left(x_{1},x_{2},t\right)\right].$$

The phase $\alpha (x_{1},x_{2},t)$ can be expressed in terms of the imaginary parts of the $\lambda_{j}$’s,

(39) $$\alpha \left(x_{1},x_{2},t\right)=\lambda_{0I}\left(t\right)+\lambda_{1I}\left(t\right)x_{1}^{2}+\lambda_{2I}\left(t\right)x_{2}^{2}+\lambda_{3I}\left(t\right)x_{1}x_{2}+\lambda_{4I}\left(t\right)x_{1}+\lambda_{5I}\left(t\right)x_{2},$$

while $R(x_{1},x_{2})$ is given by the real parts of the $\lambda_{j}$’s,

(40) $$R\left(x_{1},x_{2},t\right)=e^{2(\lambda_{0R}\left(t\right)+\lambda_{1R}\left(t\right)x_{1}^{2}+\lambda_{2R}\left(t\right)x_{2}^{2}+\lambda_{3R}\left(t\right)x_{1}x_{2}+\lambda_{4R}\left(t\right)x_{1}+\lambda_{5R}\left(t\right)x_{2})}.$$

Inserting the ansatz (35) into the nonlinear Schrödinger Equation (37) it is possible to prove, after some algebra, that (35) constitutes a time dependent solution of (37), provided that the real and imaginary parts of the $\lambda_{j}\left(t\right)$’s comply with the following set of twelve coupled ordinary differential equations,

(41) $$\begin{array}{c} \lambda_{0R}^{\prime }\left(t\right)+\frac{\hbar }{m}\left(\lambda_{1I}\left(t\right)+\lambda_{2I}\left(t\right)+\lambda_{4R}\left(t\right)\lambda_{4I}\left(t\right)+\lambda_{5R}\left(t\right)\lambda_{5I}\left(t\right)\right)=0\\ \lambda_{0I}^{\prime }\left(t\right)+\frac{\hbar }{m}\left(-\lambda_{1R}\left(t\right)-\lambda_{2R}\left(t\right)-\frac{1}{2}\lambda_{4R}^{2}\left(t\right)+\frac{1}{2}\lambda_{4I}^{2}\left(t\right)-\frac{1}{2}\lambda_{5R}^{2}\left(t\right)+\frac{1}{2}\lambda_{5I}^{2}\left(t\right)\right)+\hbar \nu \left(\lambda_{0I}\left(t\right)-\langle \alpha \rangle \left(t\right)\right)=0\\ \lambda_{1R}^{\prime }\left(t\right)+\frac{\hbar }{m}\left(4\lambda_{1R}\left(t\right)\lambda_{1I}\left(t\right)+\lambda_{3R}\left(t\right)\lambda_{3I}\left(t\right)\right)=0\\ \lambda_{1I}^{\prime }\left(t\right)+\frac{\hbar }{m}\left(-2\lambda_{1R}^{2}\left(t\right)+2\lambda_{1I}^{2}\left(t\right)-\frac{1}{2}\lambda_{3R}^{2}\left(t\right)+\frac{1}{2}\lambda_{3I}^{2}\left(t\right)\right)+\frac{1}{2}\frac{m\omega^{2}}{\hbar }+\hbar \nu \lambda_{1I}\left(t\right)=0\\ \lambda_{2R}^{\prime }\left(t\right)+\frac{\hbar }{m}\left(4\lambda_{2R}\left(t\right)\lambda_{2I}\left(t\right)+\lambda_{3R}\left(t\right)\lambda_{3I}\left(t\right)\right)=0\\ \lambda_{2I}^{\prime }\left(t\right)+\frac{\hbar }{m}\left(-2\lambda_{2R}^{2}\left(t\right)+2\lambda_{2I}^{2}\left(t\right)-\frac{1}{2}\lambda_{3R}^{2}\left(t\right)+\frac{1}{2}\lambda_{3I}^{2}\left(t\right)\right)+\frac{1}{2}\frac{m\omega^{2}}{\hbar }+\hbar \nu \lambda_{2I}\left(t\right)=0\\ \lambda_{3R}^{\prime }\left(t\right)+\frac{\hbar }{m}\left(2\lambda_{1R}\left(t\right)\lambda_{3I}\left(t\right)+2\lambda_{1I}\left(t\right)\lambda_{3R}\left(t\right)+2\lambda_{2R}\left(t\right)\lambda_{3I}\left(t\right)+2\lambda_{2I}\left(t\right)\lambda_{3R}\left(t\right)\right)=0\\ \lambda_{3I}^{\prime }\left(t\right)+\frac{\hbar }{m}\left(-2\lambda_{1R}\left(t\right)\lambda_{3R}\left(t\right)+2\lambda_{1I}\left(t\right)\lambda_{3I}\left(t\right)-2\lambda_{2R}\left(t\right)\lambda_{3R}\left(t\right)+2\lambda_{2I}\left(t\right)\lambda_{3I}\left(t\right)\right)+\hbar \nu \lambda_{3I}\left(t\right)=0\\ \lambda_{4R}^{\prime }\left(t\right)+\frac{\hbar }{m}\left(2\lambda_{1R}\left(t\right)\lambda_{4I}\left(t\right)+2\lambda_{1I}\left(t\right)\lambda_{4R}\left(t\right)+\lambda_{3R}\left(t\right)\lambda_{5I}\left(t\right)+\lambda_{3I}\left(t\right)\lambda_{5R}\left(t\right)\right)=0\\ \lambda_{4I}^{\prime }\left(t\right)+\frac{\hbar }{m}\left(-2\lambda_{1R}\left(t\right)\lambda_{4R}\left(t\right)+2\lambda_{1I}\left(t\right)\lambda_{4I}\left(t\right)-\lambda_{3R}\left(t\right)\lambda_{5R}\left(t\right)+\lambda_{3I}\left(t\right)\lambda_{5I}\left(t\right)\right)+\hbar \nu \lambda_{4I}\left(t\right)=0\\ \lambda_{5R}^{\prime }\left(t\right)+\frac{\hbar }{m}\left(2\lambda_{2R}\left(t\right)\lambda_{5I}\left(t\right)+2\lambda_{2I}\left(t\right)\lambda_{5R}\left(t\right)+\lambda_{3R}\left(t\right)\lambda_{4I}\left(t\right)+\lambda_{3I}\left(t\right)\lambda_{4R}\left(t\right)\right)=0\\ \lambda_{5I}^{\prime }\left(t\right)+\frac{\hbar }{m}\left(-2\lambda_{2R}\left(t\right)\lambda_{5R}\left(t\right)+2\lambda_{2I}\left(t\right)\lambda_{5I}\left(t\right)-\lambda_{3R}\left(t\right)\lambda_{4R}\left(t\right)+\lambda_{3I}\left(t\right)\lambda_{4I}\left(t\right)\right)+\hbar \nu \lambda_{5I}\left(t\right)=0, \end{array}$$

where

(42) $$\lambda_{jR,jI}^{\prime }\left(t\right)=\frac{d}{dt}\lambda_{jR,jI}\left(t\right).$$

The set of coupled, non-linear, first-order, ordinary differential Equation (41) need to be solved numerically. Now, $\lambda_{0R}\left(t\right)$ is determined by the normalization of the state of the system (35). So, by imposing the condition of normalization on the system, we can obtain an expression for $\lambda_{0R}\left(t\right)$ in terms of the other λ’s:

(43) $$\begin{array}{ccc} 1 & = & \int dx_{1}dx_{2}\Psi^{*}\left(x_{1},x_{2},t\right)\Psi \left(x_{1},x_{2},t\right)\\ & = & e^{2\lambda_{0R}\left(t\right)}\int dx_{1}e^{2\lambda_{1R}\left(t\right)x_{1}^{2}+2\lambda_{4R}\left(t\right)x_{1}}\int dx_{2}e^{2\lambda_{2R}\left(t\right)x_{2}^{2}+2\lambda_{3R}\left(t\right)x_{1}x_{2}+2\lambda_{5R}\left(t\right)x_{2}}. \end{array}$$

The integral appearing in the right hand side of the above equation only converges if

(44) $$\lambda_{1R}\left(t\right)<0,\;\lambda_{2R}\left(t\right)<0\;\mathrm{and}\;\lambda_{3R}^{2}\left(t\right)<4\lambda_{1R}\left(t\right)\lambda_{2R}\left(t\right).$$

Evaluating this integral and solving for $\lambda_{0R}$ gives

(45) $$\begin{array}{ccc} \lambda_{0R}\left(t\right) & = & \frac{1}{2}\ln\;\left(\frac{\sqrt{4\lambda_{1R}\left(t\right)\lambda_{2R}\left(t\right)-\lambda_{3R}^{2}\left(t\right)}}{\pi }\right)\\ & & \;+\frac{\lambda_{1R}\left(t\right)\lambda_{5R}^{2}\left(t\right)+\lambda_{2R}\left(t\right)\lambda_{4R}^{2}\left(t\right)-\lambda_{3R}\left(t\right)\lambda_{4R}\left(t\right)\lambda_{5R}\left(t\right)}{4\lambda_{1R}\left(t\right)\lambda_{2R}\left(t\right)-\lambda_{3R}^{2}\left(t\right)}. \end{array}$$

Since a wave function which is normalized at some time *t* is then automatically normalized for all time, it is sufficient to impose these conditions on the initial values of the $\lambda_{j}$’s.

Evaluating the nested integrals (see Equations (15) and (19)) in the expression for the total entanglement (9), results in the following expression:

(46) $$E\left(t\right)=1-\frac{\pi^{2}e^{4\lambda_{0R}\left(t\right)}e^{-\frac{4\left(\lambda_{1R}\left(t\right)\lambda_{5R}^{2}\left(t\right)+\lambda_{2R}\left(t\right)\lambda_{4R}^{2}\left(t\right)-\lambda_{3R}\left(t\right)\lambda_{4R}\left(t\right)\lambda_{5R}\left(t\right)\right)}{4\lambda_{1R}\left(t\right)\lambda_{2R}\left(t\right)-\lambda_{3R}^{2}\left(t\right)}}}{\sqrt{16\lambda_{1R}^{2}\left(t\right)\lambda_{2R}^{2}\left(t\right)-4\lambda_{1R}\left(t\right)\lambda_{2R}\left(t\right)\lambda_{3R}^{2}\left(t\right)+\lambda_{3I}^{2}\left(t\right)\left(4\lambda_{1R}\left(t\right)\lambda_{2R}\left(t\right)-\lambda_{3R}^{2}\left(t\right)\right)}}.$$

Substituting for $\lambda_{0R}\left(t\right)$ from (45) and simplifying leads to a final compact expression for the total entanglement of the system:

(47) $$E\left(t\right)=1-\sqrt{\frac{4\lambda_{1R}\left(t\right)\lambda_{2R}\left(t\right)-\lambda_{3R}^{2}\left(t\right)}{4\lambda_{1R}\left(t\right)\lambda_{2R}\left(t\right)+\lambda_{3I}^{2}\left(t\right)}}.$$

Notice that the entanglement only depends on $\lambda_{1R}\left(t\right),\lambda_{2R}\left(t\right),\lambda_{3R}\left(t\right)$ and $\lambda_{3I}\left(t\right)$. As $\lambda_{3I}\left(t\right)$ is squared in the total entanglement expression, the sign of $\lambda_{3I}\left(t\right)$ does not affect the total entanglement. By setting $\lambda_{3I}\left(0\right)=0$ and $\lambda_{3R}\left(0\right)=0$, the total entanglement E(t)=0 and so the state is separable and remains separable. The configuration and phase entanglement of a separable state are also zero and remain zero irrespective of the choice of parameters ν and ω. The total entanglement of the initial state can be chosen to be maximal by making the numerator inside the square root in Equation (47) as small as possible or the denominator as large as possible, which is achieved by choosing $\lambda_{3R}\left(0\right)$ such that it approaches $2\sqrt{\lambda_{1R}\left(0\right)\lambda_{2R}\left(0\right)}$ or by choosing $|\lambda_{3I}\left(0\right)|$ to be very large. This makes sense as we would expect the total entanglement of our initial state to depend strongly on $\lambda_{3}\left(0\right)$ since this is the coefficient of the cross-term in the Gaussian state (35).

**Figure 1:**
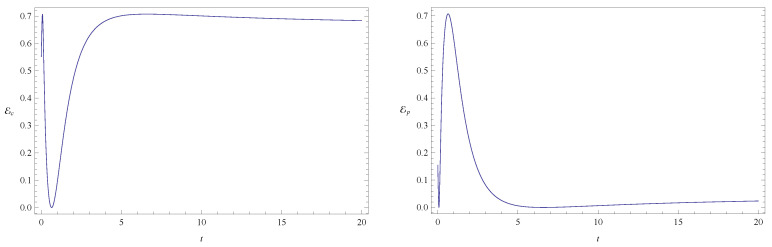
Plots of configuration entanglement $E_{c}$ (left) and phase entanglement $E_{p}$ (right) as a function of time for a free particle without friction (ν=0 and ω=0). The randomly chosen initial conditions are given in column 3 of Table 1. The total initial entanglement of the state is E=0.70717. The quantities $E_{c}$ and $E_{p}$ are dimensionless. Units of time, length and mass are chosen such that ℏ=1 and m=1.

**Figure 2:**
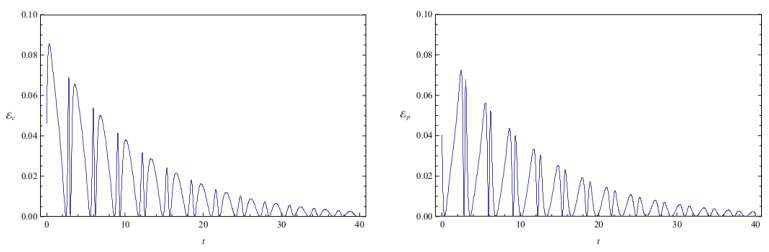
Configuration entanglement $E_{c}$ (left) and phase entanglement $E_{p}$ (right) as a function of time for an initial state of low entanglement. The initial conditions are given in column 1 of Table 1. The system parameters are ν=0.1 and ω=1, and the total initial entanglement for the state is E=0.086. The quantities are measured in the same units as in Figure 1.

**Figure 3:**
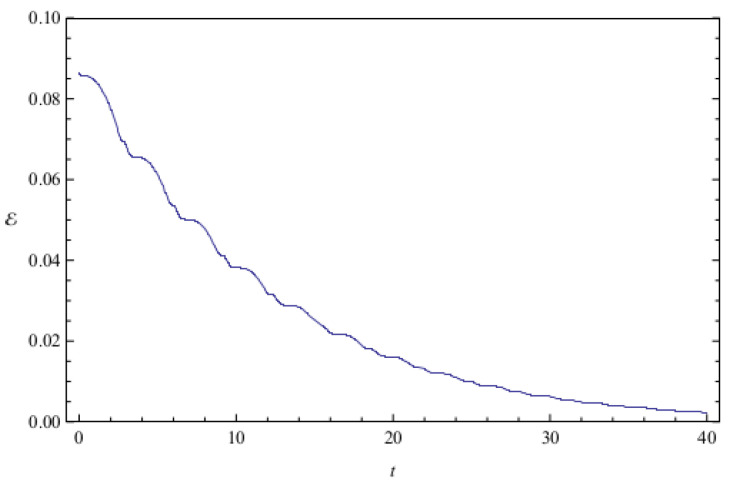
Total entanglement E as a function of time for the initial state of low entanglement considered in Figure 2. The system parameters are ν=0.1 and ω=1, and the total initial entanglement for the state is E=0.086. The quantities are measured in the same units as in Figure 1.

**Figure 4:**
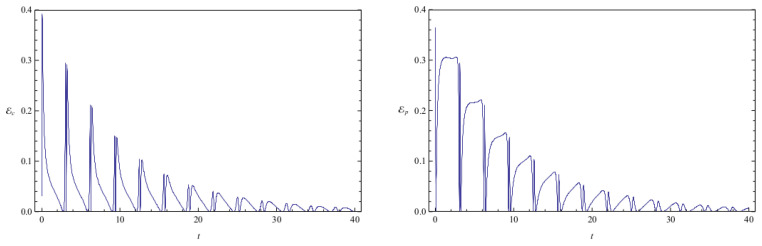
Configuration entanglement $E_{c}$ (left) and phase entanglement $E_{p}$ (right) as a function of time for an initial state of intermediate (representative) entanglement. The initial conditions are given in column 2 of Table 1. The system parameters are ν=0.1 and ω=1, and the total initial entanglement for the state is E=0.395. The quantities are measured in the same units as in Figure 1.

**Figure 5:**
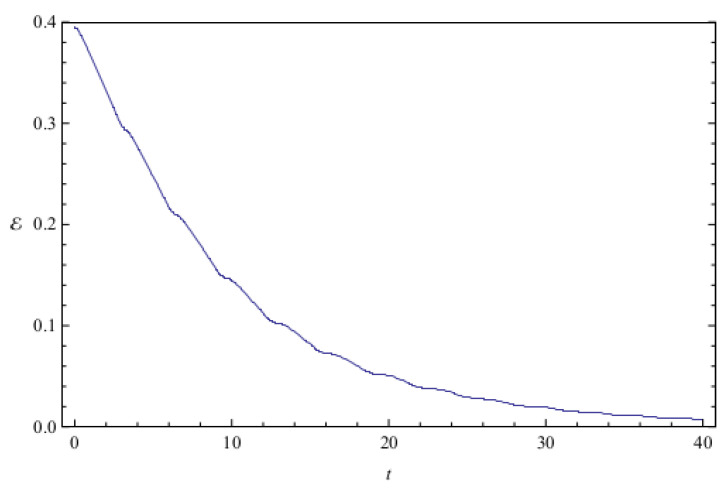
Total entanglement E as a function of time for the initial state of intermediate (representative) entanglement considered in Figure 4. The system parameters are ν=0.1 and ω=1, and the total initial entanglement for the state is E=0.395. The quantities are measured in the same units as in Figure 1.

**Figure 6:**
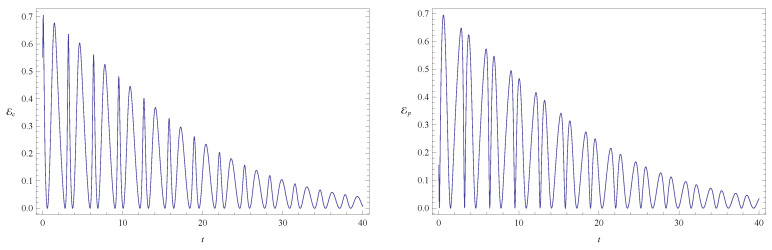
Plots of configuration entanglement $E_{c}$ (left) and phase entanglement $E_{p}$ (right) as a function of time for a randomly chosen initial state (initial conditions are given in column 3 of Table 1). The values of the physical parameters characterizing the system are ν=0.1 and ω=1, and the total initial entanglement for the state is E=0.70717. The quantities are measured in the same units as in Figure 1.

**Figure 7:**
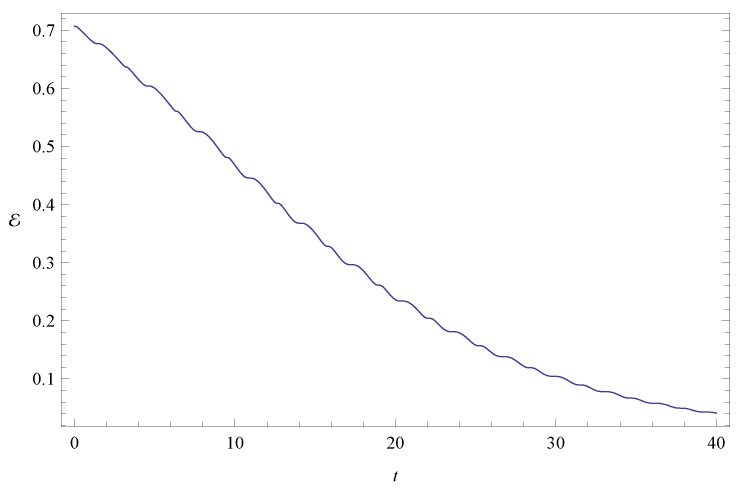
Total entanglement E as a function of time. The system parameters, initial conditions, and units used are the same as in Figure 6.

**Figure 8:**
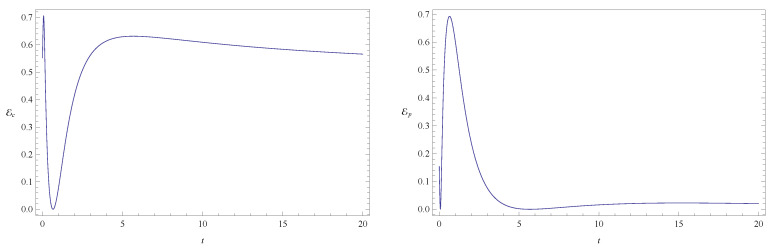
Plots of configuration entanglement $E_{c}$ (left) and phase entanglement $E_{p}$ (right) as a function of time for the same initial state as in Figure 6 and Figure 7. The values of the physical parameters characterizing the system are ν=0.1 and ω=0, and the total initial entanglement for the state is E=0.70717. The quantities are measured in the same units as in Figure 1.

**Figure 9:**
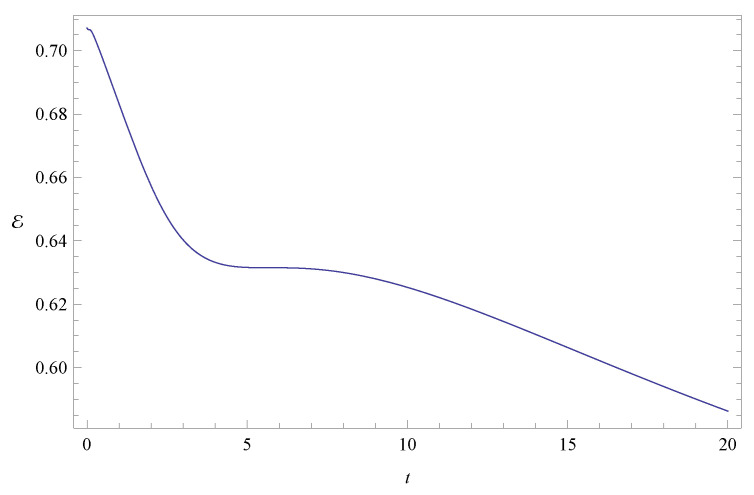
Total entanglement E as a function of time. The system parameters, initial conditions, and units used are the same as in Figure 8.

**Figure 10:**
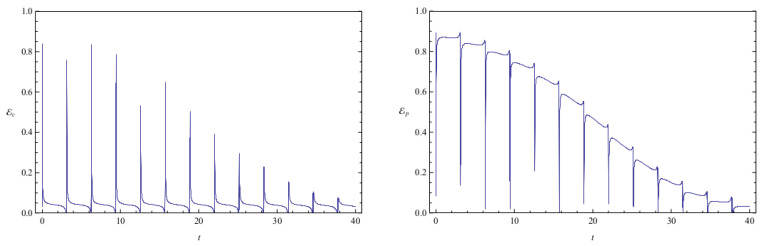
Plots of configuration entanglement $E_{c}$ (left) and phase entanglement $E_{p}$ (right) as a function of time for a highly entangled initial state (initial conditions are given in column 4 of Table 1). The system parameters are ν=0.1 and ω=1, and the total initial entanglement for the state is E=0.923.

**Figure 11:**
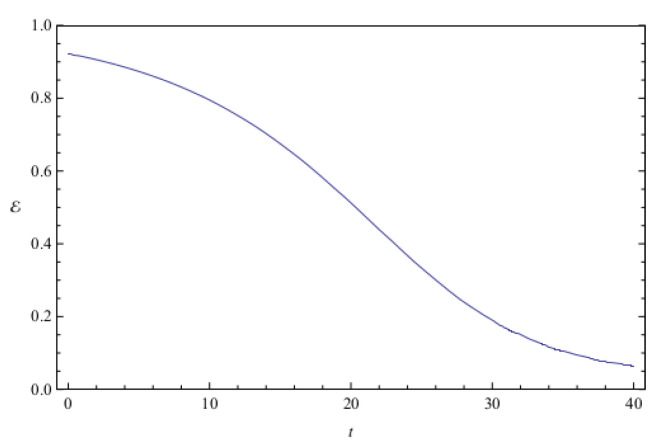
Total entanglement E as a function of time for the same highly entangled initial state considered in Figure 10. The system parameters are ν=0.1 and ω=1, and the total initial entanglement for the state is E=0.923.

We investigated numerically the evolution of the entanglement indicators for numerous initial states and for a variety of values for ν and ω. We solved numerically the equations of motion (41) for the $\lambda_{j}$’s and evaluated, on the corresponding time-dependent Gaussian wave packet, the configuration and phase entanglement indicators, $E_{c}$ and $E_{p}$. The conclusion was that the particular choices of initial states given in Table 1, and of parameters ν (ν=0 or 0.1) and ω (ω=0 or 1), are representative of the behaviour of states in general. For the initial state referred to as “generally entangled" (column 3 in Table 1) the coefficients were chosen randomly, whereas for the states with low, intermediate and high entanglement listed in Table 1, the coefficients were specifically chosen. When choosing these coefficients (randomly or otherwise) we actually choose all of them except $\lambda_{0R}$, which is calculated in terms of the other coefficients to satisfy normalization. The only difference between the coefficients of the intermediately entangled and highly entangled states is that $\lambda_{3I}\left(0\right)$ is first taken to be 10 and then 100. The time evolution of the entanglement indicators for the aforementioned initial states is illustrated in Figure 1, Figure 2, Figure 3, Figure 4, Figure 5, Figure 6, Figure 7, Figure 8, Figure 9, Figure 10 and Figure 11. Please note that for computing the numerical results displayed in the figures we have set *ℏ* and *m* to unity (i.e., we use atomic units).

As already mentioned, the time evolution of the entanglement indicators for the initial states listed in Table 1 is depicted in Figure 1, Figure 2, Figure 3, Figure 4, Figure 5, Figure 6, Figure 7, Figure 8, Figure 9, Figure 10 and Figure 11. In Figure 1 we show the evolution of the configuration and phase indicators of entanglement for an entangled state of two free particles (that is, with no confining potential) moving under no friction. In this system the total entanglement $E_{c}+E_{p}$ is conserved, although $E_{c}$ and $E_{p}$ are individually time dependent. Consistently with the fact that $E_{c}+E_{p}$ is constant in time, we see in Figure 1 that the minima of one indicator coincides to maxima of the other one (at these points one has $\left(dE_{c}/dt\right)=\left(dE_{p}/dt\right)=0$ and $\left(d^{2}E_{c}/dt^{2}\right)=-\left(d^{2}E_{p}/dt^{2}\right)$, implying that the extrema of one quantity coincide with the opposite extrema of the other one).

Figure 2 shows the time evolution of $E_{c}$ and $E_{p}$ for a pair of quantum particles evolving under the effect of friction while confined by a common harmonic potential well. The parameters characterizing the friction term and the harmonic potential are ν=0.1 and ω=1, respectively. The initial state, characterized by the coefficients appearing in the first column of Table 1, is a state of low entanglement with E=0.086. The time evolution of the total entanglement $E=E_{c}+E_{p}$ is depicted in Figure 3 for the same system and initial state as in Figure 2. The time evolution of $E_{c}$ and $E_{p}$ is depicted in Figure 4, for a pair of particles starting with an initial state of intermediate entanglement (E=0.395), for the same system parameters as in Figure 2 and Figure 3. The corresponding evolution of the total entanglement is plotted in Figure 5. The evolution of $E_{c}$ and $E_{p}$ for a pair of particles for the same system parameters as in Figure 4 and Figure 5, for a randomly chosen initial state (corresponding to the third column in Table 1) is shown in Figure 6. The evolution of the total entanglement is exhibited in Figure 7. For the same initial conditions as in Figure 6 and Figure 7, Figure 8 depicts the evolution of $E_{c}$ and $E_{p}$ for a pair of free particles (that is, with no external confining potential; ω=0) moving under the effect of friction (ν=0.1). The evolution of the total entanglement is shown in Figure 9. Finally, the evolution of $E_{c}$ and $E_{p}$ for a highly entangled initial state (E=0.923) with ν=0.1 and ω=1 is shown in Figure 10. The behavior of the corresponding total entanglement is depicted in Figure 11.

It can be appreciated in Figure 2, Figure 4, and Figure 10 that, when the particles evolve under friction in a common harmonic potential, both the configuration entanglement and the phase entanglement exhibit a strong oscillatory behaviour. The maximum values of one of the entanglement indicators tends to coincide with the minimum values of the other one. This behaviour is inherited from the corresponding behavior observed in the conservative case, which we have already discussed. This is due to the fact that, in the cases that we have studied, the time-scale of the energy dissipation is slower than the time-scale of the oscillations generated by the harmonic confining field. Consequently, during one complete harmonic period the energy stays approximately constant, and the system approximately behaves as in the conservative case.

The amplitude of the entanglement oscillations tends to decrease with time. This trend is due to the decrease in energy of the system, associated with the friction term in the nonlinear Schrödinger Equation (37). Notice that Equation (37) does not have a stochastic force term [4]. The solution of the equations of motion (41) for the λ-coefficients characterizing the evolving Gaussian wave packet, at large times, asymptotically evolves to $\lambda_{0I}=\lambda_{00}-\omega t$, $\lambda_{1R}=\lambda_{2R}=-\frac{m\omega }{2\hbar }$, $\lambda_{0R}$ given by (45), and the rest of the λ’s equal to zero. Here $\lambda_{00}$ is a dimensionless constant. This asymptotic solution corresponds to the wave function,

(48) $$\psi {\left(x_{1},x_{2},t\right)}_{\mathrm{asympt}}\;=\;\sqrt{\frac{m\omega }{\hbar \pi }}\exp\left[i\left(\lambda_{00}-\omega t\right)\right]\;\exp\left[-\frac{m\omega }{2\hbar }\left(x_{1}^{2}+x_{2}^{2}\right)\right],$$

which represents the ground state of the two particles in the harmonic potential $\frac{1}{2}m\omega^{2}\left(x_{1}^{2}+x_{2}^{2}\right)$. It can be directly verified that (48) is a solution of (37). Please note that the wave function (48) describes a separable state. This is the reason, for the system here under consideration, that both entanglement indicators $E_{c}$ and $E_{p}$ tend to zero for large times. In summary, as the system looses energy due to friction, it relaxes towards its ground state, which has no entanglement.

The total entanglement, depicted in Figure 3, Figure 5 and Figure 11, also decreases in time, but in a more smooth way, with the oscillatory features highly attenuated. We see that in this system the amount of entanglement of the two-particle state (configuration entanglement, phase entanglement, and total entanglement) tends to decrease in time due to the dissipative effects. A similar decreasing trend can be observed in Figure 8 and Figure 9, corresponding to a two-particle system experiencing friction but with no confining potential. However, in this case, where the common harmonic potential is absent, the oscillatory behaviour of $E_{c}$ and $E_{p}$ is less strong than the one exhibited in Figure 2, Figure 4, and Figure 10. The behaviour of the total entanglement in Figure 9 seems to have more structure than the corresponding behaviour in Figure 3. Some further conclusions from all the numerical investigations were that when ω≠0, then for smaller initial total entanglement the periodicity for the total entanglement is more apparent compared to higher initial values for the total entanglement. Also, as ω increases the frequency of the entanglement oscillations (total entanglement, configuration entanglement and phase entanglement) increases, as expected for an oscillating system. As ν increases, the amplitude of the entanglement oscillations decreases more rapidly, as expected when friction plays a role.

## 5. Conclusions

We revisited the concept of entanglement within the Bohmian formulation of quantum mechanics. We introduced two partial measures for the amount of entanglement corresponding to a pure state of a pair of Bohmian quantum particles. These two quantities are directly related to the main ingredients of the Bohmian dynamics, and admit a clear interpretation in terms of that dynamics. One of these measures is associated with the statistical correlations exhibited by the joint probability density in configuration space corresponding to a pair of Bohmian particles. The other partial measure corresponds to the correlations associated with the phase of the joint wave function, and describes the non-separability of the Bohmian velocity field. We refer to these two measures, respectively, as the configuration entanglement indicator and the phase entanglement indicator. The sum of these two components is equal to the total entanglement of the joint quantum state, as measured by the linear entropy of the single-particle reduced density matrix. We investigated the main properties of the configuration and the phase entanglement indicators and, as an illustrative application, explored the time evolution of these quantities, corresponding to the dissipative dynamics of an initially entangled two-particle quantum system evolving under the effect of friction. The fact that the entanglement indicators advanced here are directly defined in terms of the elements of the Bohmian formalism allows for their application to the study of entanglement in extensions or modifications of Bohm’s theory, such as the one recently advanced by Valentini [20,21], where some ingredients of the standard quantum formalism might be problematic. In the present work we have restricted our considerations to entanglement in pure states. It would be interesting to explore extensions to mixed states of the entanglement indicators explored here, although the Bohmian dynamics of mixed states is, in general, much less developed than that of pure states. Any further contributions along these or related lines of inquiry will be very welcome.

## Figures and Tables

**Table 1:** Table of initial conditions.

	Low Entanglement	Intermediately Entangled	Generally Entangled	Highly Entangled
E(0)	0.086	0.395	0.70717	0.923
$\lambda_{0R}\left(0\right)$	$\frac{1}{2}\ln\left(\frac{\sqrt{91}}{5\pi }\right)-\frac{5}{7}$	$\frac{1}{2}\ln\left(\frac{2\sqrt{15}}{\pi }\right)-\frac{1}{6}$	−122.79117	$\frac{1}{2}\ln\left(\frac{2\sqrt{15}}{\pi }\right)-\frac{1}{6}$
$\lambda_{0I}\left(0\right)$	1	1	4.21132	1
$\lambda_{1R}\left(0\right)$	−1	−4	−3.29371	−4
$\lambda_{1I}\left(0\right)$	1	1	−3.51362	1
$\lambda_{2R}\left(0\right)$	−1	−4	−0.613171	−4
$\lambda_{2I}\left(0\right)$	1	1	−0.86058	1
$\lambda_{3R}\left(0\right)$	$\frac{3}{5}$	2	−2.54191	2
$\lambda_{3I}\left(0\right)$	$\frac{3}{5}$	10	−3.28337	100
$\lambda_{4R}\left(0\right)$	1	1	7.56778	1
$\lambda_{4I}\left(0\right)$	1	1	3.99994	1
$\lambda_{5R}\left(0\right)$	1	1	−4.69103	1
$\lambda_{5I}\left(0\right)$	1	1	3.07785	1
